# Introducing a Unique Communication Method for Mental Health Crises: A Pilot Study on Staff and Patients in a Young Adult Male Psychiatric Unit

**DOI:** 10.1192/j.eurpsy.2026.10956

**Published:** 2026-07-31

**Authors:** N. Sinai, M. Kupchik, S. Loebenstein

**Affiliations:** Merhavim, Ness Ziona, Israel

## Abstract

**Introduction:**

Effective communication with patients experiencing acute psychiatric crises is crucial for preventing escalation and fostering a therapeutic alliance. A specialized communication method, developed by the Magnus organization, is designed to create a meaningful connection with patients in extreme distress, emphasizing de-escalation, strengthening trust between staff and patients, and promoting engagement with treatment programs.

*The main research question - Does implementing structured communication training program for staff improve staff self-efficacy and increase patients’ trust in staff, offering a unique perspective on both staff skills and patient experience in a male young adult psychiatric inpatient unit?*

**Objectives:**

This study aimed to evaluate the impact of implementing Magnus’ communication training program on staff self-efficacy and patients’ trust in staff in a male psychiatric inpatient unit for young adults (ages 18–30). The study offers a unique perspective by focusing not only on patients’ perceptions of trust, but also on staff members’ sense of self-efficacy, which may contribute to broader improvements in multiple aspects of clinical practice and patient care.

**Methods:**

Staff from a male young adult psychiatric unit (ages 18–30) participated in an eight-session, three-month communication training program delivered by Magnus instructors. Staff completed the **General Self-Efficacy Scale (GSE)** and the **Self-Efficacy in Patient-Centeredness Questionnaire (SE-12)** before and after the program. To assess patient trust in staff, approximately 30 patients completed the **Patient-Provider Questionnaire (PPQ)** and the **Trust in Physicians Scale (TPS)** before the intervention, and a separate group of 30 patients completed the same questionnaires after the intervention.

**Results:**

After the training, staff showed significantly higher self-efficacy (GSE, SE-12), and patient trust in staff (PPQ, TPS) also increased significantly, indicating positive effects on both staff confidence and the therapeutic relationship.

**Conclusions:**

The findings suggest that implementing an effective communication method and structured staff training can enhance functioning in a psychiatric inpatient unit on two levels: improving staff self-efficacy and strengthening patient trust in staff. Increased staff self-efficacy may indirectly contribute to higher patient trust, highlighting the importance of supporting staff skills to promote better therapeutic relationships and overall ward functioning.

**Disclosure of Interest:**

None Declared

